# Ischemic stroke of unclear aetiology: a case-by-case analysis and call for a multi-professional predictive, preventive and personalised approach

**DOI:** 10.1007/s13167-022-00307-z

**Published:** 2022-11-17

**Authors:** Olga Golubnitschaja, Pavel Potuznik, Jiri Polivka, Martin Pesta, Olga Kaverina, Claus C. Pieper, Martina Kropp, Gabriele Thumann, Carl Erb, Alexander Karabatsiakis, Ivana Stetkarova, Jiri Polivka, Vincenzo Costigliola

**Affiliations:** 1 Predictive, Preventive and Personalised (3P) Medicine, Department of Radiation Oncology, University Hospital Bonn, Rheinische Friedrich-Wilhelms-Universität Bonn, 53127 Bonn, Germany; 2 Department of Neurology, University Hospital Plzen and Faculty of Medicine in Plzen, Charles University, Prague, Czech Republic; 3 Department of Histology and Embryology, Faculty of Medicine in Plzen, Charles University, Prague, Czech Republic; 4 Department of Histology and Embryology, and Biomedical Centre, Faculty of Medicine in Plzen, Charles University, Prague, Czech Republic; 5 Department of Biology, Faculty of Medicine in Plzen, Charles University, Prague, Czech Republic; 6 Clinic for Diagnostic and Interventional Radiology, University Hospital Bonn, Rheinische Friedrich-Wilhelms-Universität Bonn, 53127 Bonn, Germany; 7 Experimental Ophthalmology, University of Geneva, 1205 Geneva, Switzerland; 8 Ophthalmology Department, University Hospitals of Geneva, 1205 Geneva, Switzerland; 9 Private Institute of Applied Ophthalmology, Berlin, Germany; 10 Institute of Psychology, Department of Clinical Psychology II, University of Innsbruck, Innsbruck, Austria; 11 Department of Neurology, University Hospital Kralovske Vinohrady, Third Faculty of Medicine, Charles University, Prague, Czech Republic; 12 European Medical Association, EMA, Brussels, Belgium

**Keywords:** Predictive preventive personalised medicine (PPPM / 3PM), Ischemic stroke, Lacunar stroke, Silent brain infarct, Health-to-disease transition, Body mass index, Sub-optimal health, Health risk assessment, Endothelial dysfunction, Hypoxia-reperfusion, Cancer, Blood–brain barrier breakdown, Metastasis, Coagulation, Pro-inflammation, Thromboembolism, Vasospasm, Endothelin-1, Flammer Syndrome phenotype, Small vessel disease, Systemic effects, Stress, Vascular stiffness, Blood pressure, Connective tissue impairments, Normal-tension glaucoma, Optic nerve degeneration, Retinal microvascular abnormalities, Diabetes comorbidities, COVID-19, Mental health, Individualised protection, Primary care, Secondary care, Pre-pregnancy check-up, Young populations, Screening, Paradigm change, Health policy

## Abstract

Due to the reactive medical approach applied to disease management, stroke has reached an epidemic scale worldwide. In 2019, the global stroke prevalence was 101.5 million people, wherefrom 77.2 million (about 76%) suffered from ischemic stroke; 20.7 and 8.4 million suffered from intracerebral and subarachnoid haemorrhage, respectively. Globally in the year 2019 — 3.3, 2.9 and 0.4 million individuals died of ischemic stroke, intracerebral and subarachnoid haemorrhage, respectively. During the last three decades, the absolute number of cases increased substantially. The current prevalence of stroke is 110 million patients worldwide with more than 60% below the age of 70 years. Prognoses by the World Stroke Organisation are pessimistic: globally, it is predicted that 1 in 4 adults over the age of 25 will suffer stroke in their lifetime. Although age is the best known contributing factor, over 16% of all strokes occur in teenagers and young adults aged 15–49 years and the incidence trend in this population is increasing. The corresponding socio-economic burden of stroke, which is the leading cause of disability, is enormous. Global costs of stroke are estimated at 721 billion US dollars, which is 0.66% of the global GDP.

Clinically manifested strokes are only the “tip of the iceberg”: it is estimated that the total number of stroke patients is about 14 times greater than the currently applied reactive medical approach is capable to identify and manage. Specifically, lacunar stroke (LS), which is characteristic for silent brain infarction, represents up to 30% of all ischemic strokes. Silent LS, which is diagnosed mainly by routine health check-up and autopsy in individuals without stroke history, has a reported prevalence of silent brain infarction up to 55% in the investigated populations. To this end, silent brain infarction is an independent predictor of ischemic stroke. Further**,** small vessel disease and silent lacunar brain infarction are considered strong contributors to cognitive impairments, dementia, depression and suicide, amongst others in the general population. In sub-populations such as diabetes mellitus type 2, proliferative diabetic retinopathy is an independent predictor of ischemic stroke.

According to various statistical sources, cryptogenic strokes account for 15 to 40% of the entire stroke incidence. The question to consider here is, whether a cryptogenic stroke is fully referable to unidentifiable aetiology or rather to underestimated risks. Considering the latter, translational research might be of great clinical utility to realise innovative predictive and preventive approaches, potentially benefiting high risk individuals and society at large.

In this position paper, the consortium has combined multi-professional expertise to provide clear statements towards the paradigm change from reactive to predictive, preventive and personalised medicine in stroke management, the crucial elements of which are:

- Consolidation of multi-disciplinary expertise including family medicine, predictive and in-depth diagnostics followed by the targeted primary and secondary (e.g. treated cancer) prevention of silent brain infarction
- Application of the health risk assessment focused on sub-optimal health conditions to effectively prevent health-to-disease transition
- Application of AI in medicine, machine learning and treatment algorithms tailored to robust biomarker patterns
- Application of innovative screening programmes which adequately consider the needs of young populations

## Plain English abstract

Stroke is a severe brain disease which has reached an epidemic scale worldwide: in 2019, the global stroke prevalence was 101.5 million people. The World Stroke Organisation predicted that globally, 1 in 4 adults over the age of 25 will get a stroke in their lifetime. Not only old people but also teenagers and young adults are affected. Current global costs of stroke are estimated at 721 billion US dollars. Due to undiagnosed so-called “silent” brain infarction, the number of affected individuals is about 14 times greater in the population than clinically recorded. If it remains untreated, silent brain infarction may cause many severe and fatal disorders such as dementia, depression and even suicide. In this position paper, the consortium describes how the rudimental approach to treating severely diseased people could be replaced by an innovative predictive and preventive one to protect people against the health-to-disease transition.

## Global epidemics of stroke: status quo and prognoses in figures

Due to the reactive medical approach applied to disease management, stroke has reached epidemic scale worldwide. In 2019, the global stroke prevalence was 101.5 million people, of which 77.2 million (about 76%) suffered from ischemic stroke; 20.7 and 8.4 million suffered from intracerebral and subarachnoid haemorrhage, respectively [1]. Globally in the year 2019 — 3.3, 2.9 and 0.4 million individuals died of ischemic stroke, intracerebral and subarachnoid haemorrhage, respectively.

During the last three decades, the number of cases has increased substantially. The World Stroke Organization (WSO) reported an increase in stroke incidence of 70.0%, death from stroke of 43.0%, stroke prevalence of 102.0%, and disability-adjusted life-years lost of 143.0% [2]. The current prevalence of stroke is 110 million patients worldwide with more than 60% below the age of 70 years with the highest rates of ischemic stroke recorded for the high-income countries in North America, the Middle East and Southeast Asia, amongst others [3]. Prognoses presented by the WSO are pessimistic: globally, 1 in 4 adults over the age of 25 is predicted to suffer a stroke in their lifetime [3]. Although age is the best known contributing risk factor, over 16% of all strokes occur in teenagers and young adults aged 15–49 years with increased incidence trend. The socio-economic burden of stroke is enormous since stroke is the leading cause of disability. Current global costs of stroke are estimated at 721 billion US dollars which is 0.66% of the global GDP [2].

## Clinically manifested strokes is only the “tip of the iceberg”

In 2003, Leary and Saver published evidence-based estimates of the annual incidence of first silent stroke in the USA, which indicated that for the year 1998 there were about 11 million first-ever silent MRI brain infarcts (SBI, called also “covered brain infarction”) or haemorrhages versus 770,000 symptomatic clinically manifested stroke cases [4]. These data indicate that clinically manifested strokes contribute a noticeable but minor share of the entire issue of brain infarction, which is estimated to be about 14 times greater in the population than the currently applied reactive medical approach, per definition [5], and is capable to identify and manage. Specifically, lacunar stroke (LS), being also characteristic for SBI, represents up to 30% of all ischemic strokes. Silent LS is frequently diagnosed during routine health check-ups as well as during autopsy in otherwise healthy individuals without stroke history. Depending on the focus of the study and patient stratification criteria, the reported prevalence of SBI/silent LS is 6 to 55% in the investigated populations [6–14]. Here, it is worth noting that SBI is more prevalent in females whereas clinically manifested stroke is reported to be more prevalent in males [6, 15–19]. There are obvious sex differences in the symptomatic interpretation of Cerebrovascular Diseases (CVD) and cerebrovascular infarcts, which has been traditionally considered more prevalent in the population and, therefore, described more precisely. Consequently, female-specific symptoms might be simply overlooked or interpreted wrongly resulting in poorer outcomes [20]. Further, SBI-related prevalence data are lacking for young populations [6] significantly hindering targeted primary prevention and effective protection of young populations.

## Silent brain infarction as an indicator of associated systemic disorders — the prominent examples and pathomechanisms behind the health-to-disease transition

*“…silent strokes aren’t so silent”* said Dr. Gorelik who every day examines people with abnormal brain scans indicating a silent stroke — see “Silent strokes’ found accidentally need treatment” by American Heart Association News [21] to prevent severe pathologies linked to subtle changes indicated by SBI.

### Prominent examples

#### SBI as an independent marker for ischemic stroke risks

Existing SBI strongly predicts a next silent infarction [22] and indicates the highest risk of ischemic stroke in affected individuals. Amongst the selected patient cohorts, the prevalence of SBI in the ischemic stroke patients was 57% [23].

#### Cognitive impairment and dementia

The onset of cognitive impairment and dementia are leading consequences of stroke. In a cohort of Japanese Alzheimer’s disease patients, every 3^rd^ patient was co-diagnosed with SBI [24]. Similar observations have been made from autopsies of individuals with a history of dementia [25–27]. Small vessel disease and lacunar brain infarctions are considered strong contributors to cognitive impairments and dementia in the general population [28]. In particular, SBI localised in the thalamus negatively affects cognitive tasks such as short-term memory performance [29–31], whereas non-thalamic SBI correlates with a decline in psychomotor speed tasks [23].

#### Affective disorders and suicide

Significant data indicate that SBIs below 3 mm are associated with symptoms of depression; in fact, SBI can be diagnosed in up to 50% of elderly people with major depression [23, 32–34]. Bipolar disorder type II with a suicide attempt, secondary to a lacunar state has been reported [35].

#### Migraine

Significant associations were found between migraine attacks and silent brain infarction [36, 37]. Females are 3 times more likely to be affected by migraine; high oestrogen levels increase risks of migraine with aura, thromboembolism, secondary ischemia-induced aura and ischemic stroke [38].

#### Normal-tension glaucoma

Silent cerebral infarction (SCI) has been proposed to be an independent risk factor for progression of visual field loss in patients with normal-tension glaucoma (NTG) [39]. The correlation between SCI and NTG is considered indicative for small vessel diseases, a major contributor to the NTG pathology, microvascular dysregulation and endothelial dysfunction characteristic for the NTG patient cohort and NTG-specific type of nerve degeneration resulting in progressive visual field loss. Vascular geometry may affect perfusion efficiency and the functional link between SCI, small vessel disease and normal-tension glaucoma [40].

## Pathomechanisms behind the health-to-disease transition — a vicious circle of the stroke development

A better understanding of the mechanisms underlying the health-to-disease transition in stroke is key point for cost-effective health risk assessment, predictive diagnostics, patient stratification and targeted prevention which might be offered to individuals at risk. Stroke development shares several risk factors, e.g., affected metabolic pathways and molecular targets with other stress-related diseases such as cancers and diabetes mellitus type 2 as detailed below.

### Systemic effects of the reciprocal cancer-stroke promotion: what comes first?

Cancer-associated ischemic stroke is well-described in the literature: As reported recently, cancer pathomechanisms are functionally linked to endothelial dysfunction, coagulation abnormalities, activated pro-inflammation, and platelets adhesion, which collectively increase the risk of thromboembolism and ischemic stroke [41]. Additionally, vascular toxicity and secondary dysfunction are adverse effects of current anti-cancer therapeutics. It is also to be noted that stroke survivors are diagnosed with cancer at almost double the rate of the general population [42]*.* Though, a study dedicated to the cancer incidence in young adults suffering from ischemic stroke demonstrated median time from pre-stroke cancer to stroke of about 4.9 (1.0–9.5) years, whereas from stroke to post-stroke cancer by 6.7 (2.7–10.9) years [43]. Thus, the question “*What comes first – cancer or stroke?”* has not yet been clarified. However, per evidence, both stress-related diseases share several risk factors, affected metabolic pathways and molecular targets, namely:

- Oxidative stress
- Disturbed microcirculation
- Endothelial dysfunction (ET-1)
- Compromised mitochondrial health
- Pro-inflammation
- Connective tissue impairments
- Extensive tissue remodelling, amongst other,

collectively leading to the vicious cycle in the development of the systemic hypoxia-reperfusion, ischemic lesions, activated MMPs, blood brain barrier (BBB) breakdown, brain infarction and metastases [44–48].

**Fig. 1:**
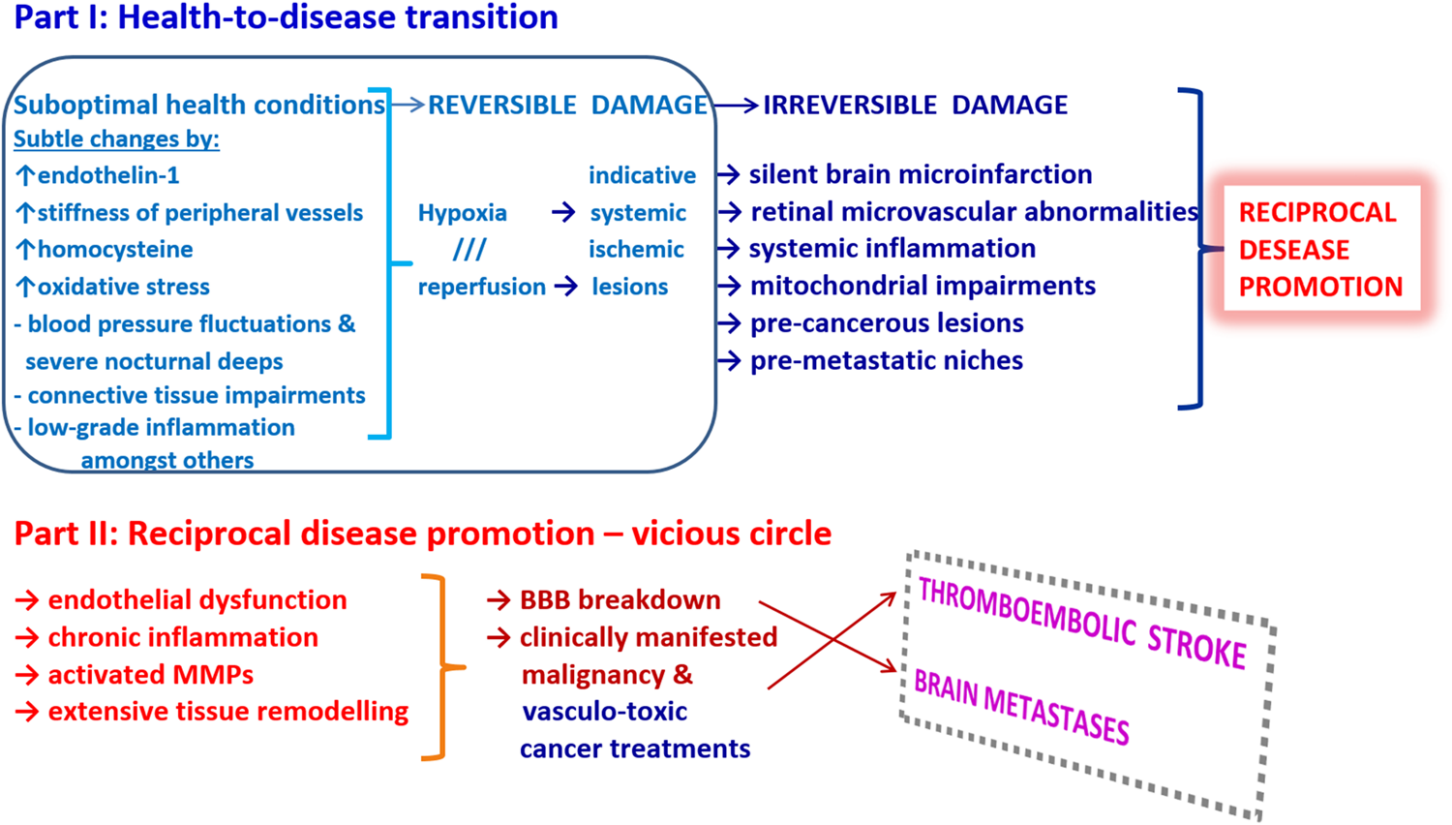
Schematic presentation of the health-to-disease transition in the stroke development and reciprocal cancer-stroke disease promotion; blood–brain barrier, BBB; and metalloproteinases, MMPs. Sub-optimal health conditions representing reversible damage are relevant for the cost-effective primary healthcare (blue frame) and are based on the individualised health risk assessment and targeted primary prevention. At this stage, subtle changes may include imbalanced stress, enhanced endothelin-1 blood plasma levels indicating pronounced vasoconstriction of peripheral vessels, increased stiffness of peripheral vessels co-diagnosed with connective tissues deficits/disease as demonstrated in pregnant women with the Flammer syndrome [45–47, 51], enhanced homocysteine levels in blood plasma potentially leading to small vessel disease and associated SBI [52], blood pressure fluctuations with remarkable nocturnal lows [6] and low-grade inflammation [53, 54], all relevant to the manifestation of hypoxia-reperfusion and systemic ischemic lesions. These changes may result in irreversible damage leading to SBI, retinal microvascular changes, systemic inflammation, mitochondrial impairments, pre-cancerous lesions and pre-metastatic niches – altogether leading to the reciprocal cancer-stroke promotion in a “vicious cycle”. To this end, thromboembolic stroke is frequently observed in (treated) patients with cancer diagnoses. In turn, brain metastases are characteristic, e.g., for patients diagnosed with the triple-negative breast cancer and the Flammer syndrome phenotype demonstrating extensive vasoconstriction and systemic hypoxic-ischemic lesions including SBI [48, 55]. Extensive tissue remodelling plays a key role in both, neurodegeneration (stroke) and metastatic cancer performed by the core of metalloproteinases, which are excellent indicators and pathology-associated biomarkers [49, 56–60]

However, by far not every cancer patient develops stroke and not all patients with a history of stroke are at high risk of cancer. Indeed, patients with brain malignancies and cancers spreading metastases to the brain such as triple-negative breast cancer [49] are at high risk of stroke as well as cancer patients with secondary vascular dysfunction who underwent vasculo-toxic therapies causing thromboembolic type of stroke [41]. On the other hand, subtle systemic hypoxic-ischemic effects causing low-grade inflammation have been proposed to be involved in the development of particularly aggressive metastatic cancers [48] the pathomechanisms of which are considered to be similar to those of non-healing wounds [50]. Consequently, for this type of cancer, SBI diagnostics might be particularly useful to indicate associated systemic effects — the concept to be further considered by the follow-up studies. Figure 1 summarises facts and hypotheses involved in the reciprocal cancer-stroke promotion.

### Diabetic retinopathy is indicative for the stroke risk

Due to pronounced vascular ageing, individuals affected by diabetes mellitus (DM) have twice the risk of stroke compared to non-diabetics [61]. Microvascular dysfunction is characteristic for DM. Hyperglycaemia, obesity, insulin resistance and hypertension have been described as the main drivers of DM-related microvascular dysfunction. Cerebral microvascular dysfunction is present already in pre-diabetic stages suggesting that cerebral microvascular disease is an early step in the overall cascade of DM pathophysiology predisposing to lacunar and haemorrhagic stroke, cognitive dysfunction, and depression [62]. For pre/diabetes care, it is crucial, to stratify patients at high versus low risk of stroke based on highly specific biomarker patterns, including the presence of diabetic retinopathy (DR), which has been associated with an increased risk of life-threatening systemic vascular complications, including stroke, coronary heart disease, and heart failure [63]. Due to the particularly high sensitivity of retinal cells to oxidative stress, retinal health provides valuable insights into patients’ risk of future vascular disease and death. Early DR manifestation is associated with an increased risk of stroke in DM. This correlation is particularly robust for DM type 2, suggesting that DR is an important biomarker for the prediction of stroke in this patient cohort [64]. Specifically, proliferative DR has been proposed as the biomarker for accelerated promotion of CVD complication in DM [65]. In fact, increased severity of DR carries a heightened risk for cerebrovascular accidents, myocardial infarction, congestive heart failure and severity of the corresponding disease outcomes in patients with type 2 DM [66].

## Cryptogenic strokes: unidentifiable aetiology or underestimated risks?

According to different statistical sources, cryptogenic strokes (CSs) account for 15 to 40% of the stroke incidence [67]. A generally accepted definition for CSs currently does not exist, although several classification systems have been provided, namely the Trial of Org 10,172 in Acute Stroke (TOAST) that defines CSS as a stroke of undetermined aetiology which is not caused by large artery atherosclerosis, cardioembolism, and small vessel occlusion. The Causative Classification of Stroke System (CCS) is a computerised algorithm determining causative and phenotypic stroke subtypes and detecting uncommon and undetermined causes including incomplete evaluation and cryptogenic stroke, and ASCOD (Atherosclerosis, Small Vessel Disease, Cardiac Causes, Other, and Dissection), which grades the likelihood that the disease was causally related to the stroke: from 0 = absence of the disease and 1 = potentially causal up to 9 = insufficient workup to rule out the disease [67]. Despite the difference between individual classification systems, the main concept is similar, namely, cryptogenic stroke is an ischemic stroke with no identifiable aetiology based on a diagnosis of exclusion.

Contextually, the question does arise, whether cryptogenic stroke is fully referable to unidentifiable aetiology or rather to underestimated risks, which have been demonstrated by recent studies but still not taken into account when considering the causes of CS, e.g., in the computerised algorithms for the automatic disease recognition, causality and phenotyping, pre-stages, corresponding biomarker patterns, etc. Considering the latter, translational research might be of great clinical utility to realise innovative predictive and preventive approaches, potentially benefiting individuals at high risk and society at large. This concept may be illustrated with the stroke cases analysed below.

## Ischemic stroke case-by-case

### Case 1: Young ischemic stroke associated with cancer

A 49-year-old woman (BMI = 16.22 kg/m2) was referred to the hospital due to a sudden onset of left-sided hemiplegia. The patient has generalisation of endometrial adenocarcinoma (pT2pN1 (3/45) M0, G2, R0 treated from 2018 with radiotherapy and chemotherapy, FIGO IIIC2, paclitaxel/carboplatina). Brain metastases were irradiated with Leksell gamma knife in 2019. The patient underwent outpatient oncological treatment. Multimodal CT of the brain revealed MCA/ACA occlusion (T-occlusion) right-sided. The patient was treated with intravenous thrombolysis (Actilyse) and mechanical thrombectomy. Re-canalisation TICI 2c was achieved and neurological findings improved to moderate left-sided hemiparesis. Later, there was a progression of the oncological disease and the patient died 10 months later due to the generalisation of endometrial adenocarcinoma in Hospic, mRS 6.

The cause of stroke: MCA/ACA occlusion (T-occlusion) is most likely a pro-coagulant condition caused by cancer — endometrial adenocarcinoma (see Fig. 1).

### Case 2: Young ischemic stroke associated with the pronounced Flammer syndrome phenotype and severe course after SARS-CoV-2 infection

A 37-year-old woman (BMI = 23.94 kg/m2) was referred to the hospital due to the onset of vision problem and clumsiness of the left limbs. At admission, left-sided homonymous hemianopsia and left-sided hemiataxia were detected. Multimodal CT revealed an acute ischaemic lesion in the left cerebellar hemisphere (penumbra without core), CT chest/lung confirmed viral pneumonia compatible with SARS-CoV-2 infection confirmed by the corresponding PCR test. Further, the patient demonstrated the Flammer syndrome phenotype with pronounced vascular maladaptation, migraine with accompanying symptoms, low BMI at young age, suboptimal sleep patterns, a tendency to low blood pressure and specific psychosocial behavioural patterns such as a pronounced meticulous personality, amongst other signs and symptoms. This is of special interest as the recently emerging field of affective immunology addresses the multidirectional-communication between emotion regulation, personality traits and immune functioning, mainly focusing on the association between pro-inflammatory signalling and free radical production by the immune system and personality traits. Upcoming findings will enable a more detailed evaluation of these processes and their interplay for their contribution and importance in the context of prediction and prevention also in ischemic stroke.

The patient was treated with intravenous thrombolysis (Actilyse) with excellent result. Other examinations: normal transthoracic and transesophageal echocardiography and ECG Holter, negative thrombotic panel, negative panel of autoimmune diseases, negative test for Fabry disease. Diffusion-weighted MRI scans 1 month after stroke onset showed no infarction. Full recovery of neurological deficit, mRS 0.

The cause of stroke: ischaemic lesion in the left cerebral hemisphere is most likely a pro-coagulant condition caused by a combination of the strongly pronounced Flammer syndrome phenotype and severe course of the SARS-CoV-2 infection.

### Case 3: Young ischemic stroke associated with the pronounced Flammer Syndrome phenotype and stress overload

A 45-year-old woman (BMI = 24.8 kg/m2) was referred to hospital due to a sudden onset of stand and gait ataxia and hypestesia on the left side of her face. Multimodal CT of the brain at admission was normal. MRI revealed an ischaemic lesion (6 × 5 × 4,5 mm) on the left side of the medulla oblongata. Recanalization therapy was not used due to exceeded time window, the patient was treated with acetylsalicylic acid and rosuvastatin, crystaloids and rehabilitation. In the past medical history, her premature birth (birth weight 1350 g) was recorded. Further, the patient demonstrated the Flammer syndrome phenotype with migraine, vascular maladaptation, thermal dysregulation, extremely low BMI at young age, Sicca syndrome and dehydration, suboptimal sleep patterns, dysmenorrhea, a tendency to low blood pressure and specific psychosocial behavioural patterns such as a pronounced meticulous personality, amongst other signs and symptoms. The patient is a smoker. Further, she reported exposure to enormous stress several months before stroke onset. Results of genetic testing showed Leiden heterozygosity and negative Fabry disease test. There were normal transthoracic and transesophageal echocardiography and ECG Holter (7 days). The patient was discharged with residual hemiataxia and instability, disability pension, mRS 3.

The cause of stroke: ischaemia in the medulla oblongata is most likely a pro-coagulant condition caused by a combination of the pronounced Flammer syndrome phenotype and stress overload which, however, has to be objectively measured.

## Predictive phenotyping and targeted primary prevention of ischemic stroke are feasible: a detailed case presentation

At the end of 2021, a 59-year-old woman underwent commercially available and internationally validated predictive diagnostic test based on mitochondrial health quality measurements (check-point and consultation by responsible general practitioner specialised in family medicine). Reason for the test was diagnosis of causes of sub-optimal health condition the patient worried about. In the patient’s medical history, the following information was considered highly relevant:

- Ischemic stroke in the family history (thrombotic vein occlusion of mother at the age of 77 years and lacunar stroke of father at the age of 88 years);
- Pronounced Flammer syndrome phenotype of the patient including low body mass index, low blood pressure, migraine with aura, tinnitus, strong vasospastic reactions under stress conditions accompanied with a significantly increased endothelin-1 level (3.2 pg/ml) in blood serum, retinal ischemic lesions diagnosed early in life (35 years of age; check-point and consultations by specialised ophthalmological clinic), and connective tissue impairments, amongst others;
- As a carrier of the Flammer syndrome phenotype, the patient is a highly stress-sensitive person that is further aggravated by her meticulous personality and permanent work/stress overload caused by a highly ambitious international academic career of the patient.

Indeed, the predictive health quality test performed, demonstrated characteristic non-compensated stress overload patterns which, in the specific case of this patient reflect ischemic-reperfusion episodes associated with vasospastic reactions of peripheral vessels and increased endothelin-1 levels [68]. The following recommendations were provided to the patient:

1. Treatment with commercially available scavenges as bioactive protection against acute stress such as emotional and psychological stress situations [68–70]
2. Evaluation of single stressors to apply individualised therapeutic approaches [71, 72]
3. Brain MRI was recommended to check for SBI as the most reliable biomarker for ischemic stroke prediction.

Follow-up report.

**Fig. 2:**
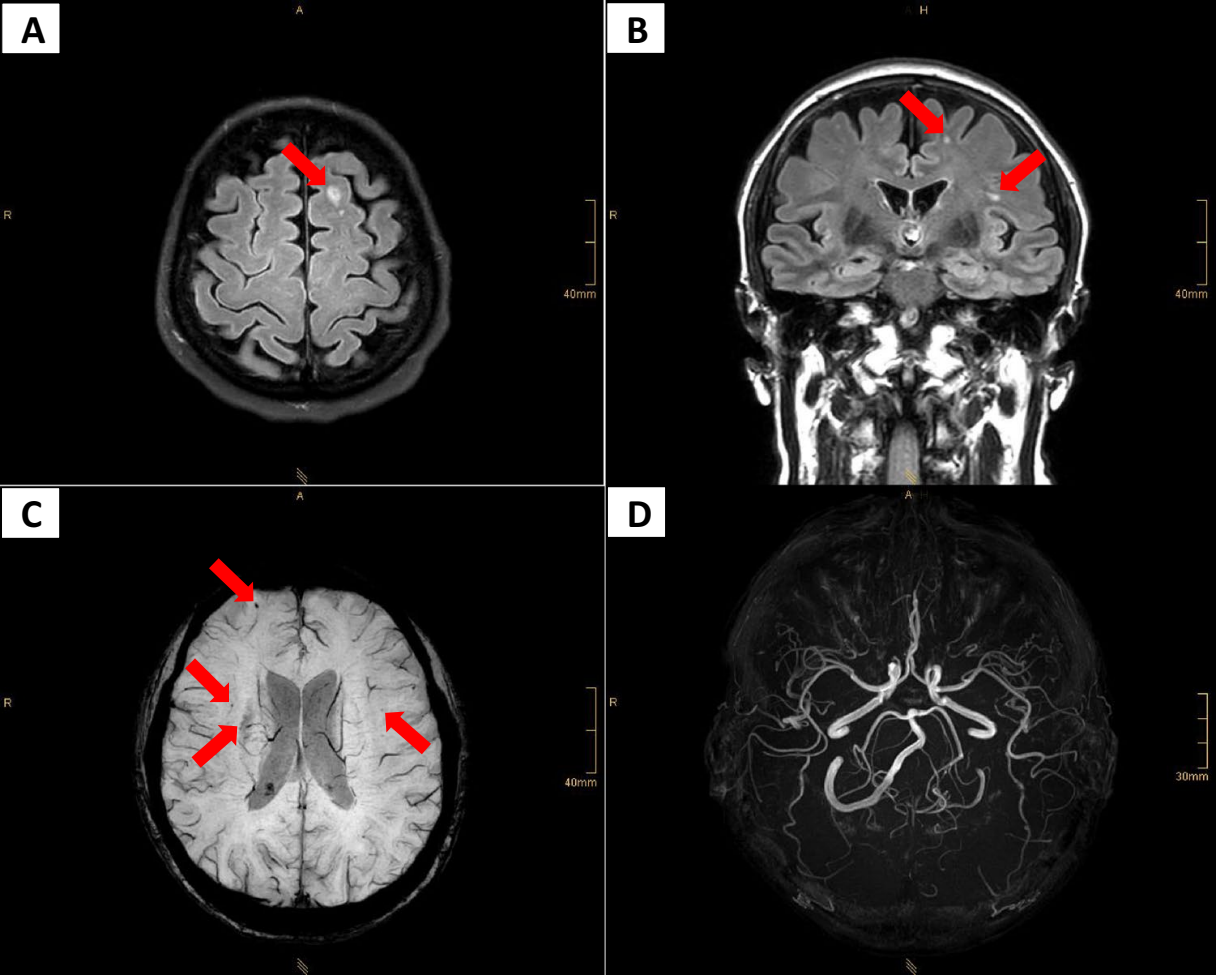
Brain-MRI performed in August 2022; **Methodology:** The examination was performed on the 3Tesla-MRI with weight-adjusted intravenous contrast medium administration (CMA, gadolinium). Sequences obtained: FLAIR*,* axial scan; DWI*,* ADC map, axial scan*;* SWI axial scan; axial FLAIR 3D scan; Time-of-Flight MR angiography; axial T2tse scan; 3D T1*-*weighted sequences after CMA with coronary and axial reconstructions. **Results and conclusions:** Although the cerebral vasculature is well-structured and intact without any detectable pathological changes (D), small vessels clearly show signs of micro-angiopathy; several lacunar microinfarction zones, white matter hyper-intensities and micro-haemorrhages (A, B, C) are evident; no any recent ischemic lesions have been recorded

- The patient is taking orally L-cysteine (500 mg, Gall Pharma) as prophylaxis against expected stress situations.
- The patient is taking ginkgo biloba (120 mg) and magnesium as daily nutritional supplement to improve microcirculation and to protect small vessels against inappropriate vasoconstriction.
- Brain MRI (check-point and consultations by the specialised diagnostic radiology unit) performed according to the recommendations has confirmed the predicted SBI (Fig. 2). However, given successful mitigation measures, no recent ischemic lesions have been detected. The current health condition of the patient is considered stabilised.

## Conclusions in the framework of 3P medicine

Due to the reactive medical approach applied to the disease management, stroke reached an epidemic scale worldwide, and current WSO prognoses are pessimistic: globally, 1 in 4 adults over the age of 25 years is predicted to get diseased on stroke in their lifetime. Moreover, the incidence trends to increase particularly for young stroke patients below 50 years of age. The corresponding socio-economic burden is enormous. Further, clinically manifested strokes are only the “tip of the iceberg”: apparently, the total size is about 14 times greater in the population than the currently applied reactive medical approach, per definition is capable to identify and manage. Specifically, the lacunar stroke builds up to 30% of all ischemic strokes being also characteristic for silent brain infarction. Silent LS is diagnosed mainly by routine health check-up and autopsy in individuals without stroke history: the reported prevalence of silent brain infarction is 6 to 55% in the investigated populations. To this end, silent brain infarction is an independent predictor of ischemic stroke. There are significant sex and age differences in symptomatic interpretation of the silent brain infarction: female sex and youth-specific symptoms are frequently overlooked or wrongly interpreted that significantly hinders targeted primary prevention and effective protection of females and young populations. Further, small-vessel disease and silent lacunar brain infarctions are considered strong contributors to cognitive impairments, dementia, depression and suicide, amongst others in the general population. In sub-populations such as diabetes mellitus type 2, specifically proliferative DR is an independent predictor of the ischemic stroke.

According to different statistical sources, cryptogenic strokes account for up to 40% of the entire stroke incidence. The question does arise whether cryptogenic stroke is fully referable to unidentifiable aetiology or rather to underestimated risks. Considering the latter, translational research might be of great clinical utility to realise innovative predictive and preventive approaches, potentially benefiting at high-risk individuals and society at large.

In this position paper, the consortium involved has combined multi-professional expertise to provide clear statements towards the paradigm change from reactive to predictive, preventive and personalised medicine in stroke management, the crucial elements of which are the following:

- Consolidation of multi-disciplinary expertise including family medicine, predictive and in-depth diagnostics followed by the targeted primary and secondary (e.g. treated cancer) prevention of the silent brain infarction
- Application of the health risk assessment focused on sub-optimal health conditions to effectively prevent health-to-disease transition
- Application of AI in medicine, machine learning and treatment algorithms tailored to robust biomarker patterns
- Application of innovative screening programmes considering the needs of young populations

## Abbreviations

*ACA*: Anterior cerebral artery
*ADC*: Apparent diffusion coefficient
*AI*: Artificial intelligence
*ASCOD*: Atherosclerosis, small vessel disease, cardiac causes, other, and dissection
*BBB*: Blood brain barrier
*BMI*: Body mass index
*CCS*: Causative classification of stroke system
*CMA*: Contrast medium administration
*CSs*: Cryptogenic strokes
*CT*: Computed tomography
*CVD*: Cerebrovascular diseases
*DM*: Diabetes mellitus
*DR*: Diabetic retinopathy
*DWI*: Diffusion-weighted imaging
*ECG*: Electrocardiogram
*ET-1*: Endothelin-1
*FIGO*: International federation of gynecology and obstetrics
*FLAIR*: Fluid attenuated inversion recovery
*GDP*: Gross domestic product
*LS*: Lacunar stroke
*MCA*: Middle cerebral artery
*MMPs*: Matrix metalloproteinases
*MRI*: Magnetic resonance imaging
*mRS*: Modified Rankin scale
*NTG*: Normal-tension glaucoma
*PCR*: Polymerase chain reaction
*PPPM/3PM*: Predictive preventive personalised medicine
*SBI*: Silent brain infarcts
*SCI*: Silent cerebral infarction
*SWI*: Susceptibility weighted imaging
*TICI*: Thrombolysis in cerebral infarction scale
*TOAST*: The trial of org 101072 in acute stroke treatment
*WSO*: World stroke organisation
